# SARS-CoV-2 transmission in schools: An updated living systematic review (version 2; November 2020)

**DOI:** 10.7189/jogh.11.10004

**Published:** 2021-06-10

**Authors:** Wei Xu, Xue Li, Yijia Dong, Marshall Dozier, Yazhou He, Amir Kirolos, Zhongyu Lang, Catherine Mathews, Nandi Siegfried, Evropi Theodoratou, UNCOVER

**Affiliations:** 1 School of Public Health and the Second Affiliated Hospital, Zhejiang University, Hangzhou, China; 2 Centre for Global Health Research, Usher Institute, University of Edinburgh, Edinburgh, United Kingdom; 3 Information Services, University of Edinburgh, Edinburgh, United Kingdom; 4 Institute of Translational Medicine, University of Liverpool, Liverpool, United Kingdom; 5 Health Systems Research Unit, South African Medical Research Council, Francie Van Zijl Drive, Parow, South Africa; 6 Cancer Research UK Edinburgh Centre, Medical Research Council Institute of Genetics and Molecular Medicine, University of Edinburgh, Edinburgh, United Kingdom

## Abstract

**Background:**

Better understanding of SARS-CoV-2 transmission risks is needed to support decision-making around mitigation measures for COVID-19 in schools.

**Methods:**

We updated a living systematic review and meta-analysis to investigate the extent of SARS-CoV-2 transmission in schools. In this update we modified our inclusion criteria to include: 1) cohort studies; 2) cross-sectional studies that investigated and cross-assessed SARS-COV-2 positivity rates in schools and communities; and 3) pre-post studies. We performed risk of bias evaluation for all included studies using the Newcastle-Ottawa Scale (NOS).

**Results:**

6270 articles were retrieved and six new studies were added in this update. In total from the two updates and using the new inclusion criteria, we identified 11 cohort studies (1^st^ update: n = 5; 2^nd^ update: n = 6) and one cross-sectional study (1^st^ update: n = 1; 2^nd^ update: n = 0). We performed a meta-analysis on nine of the 11 cohort studies investigating IAR in schools. Nine cohort studies reported a total of 91 student and 52 staff index cases that exposed 5698 contacts with 101 secondary infections (overall infection attack rate (IAR) = 1.45%, 95% CI = 0.31%-3.26%). IARs for students and school staff were 1.66% (95% CI = 0.08%-4.78%) and 1.18% (95% CI = 0.00%-4.43%) respectively. The risk of bias was found to be high for most studies identified, limiting the confidence in results.

**Conclusions:**

There is limited high-quality evidence available to quantify the extent of SARS-CoV-2 transmission in schools or to compare it to community transmission. Emerging evidence suggests the overall IAR and SARS-CoV-2 positivity rate in school settings are low. Higher IAR were found in students, compared to staff.

**Note:**

This article is a living systematic review that will be updated to reflect emerging evidence. This is the second version of the original article published on 23 December 2020 (J Glob Health 2020;11:021104), and previous versions can be found as data supplements. When citing this paper please consider adding the version number and date of access for clarity.

In response to the COVID-19 pandemic, 107 countries implemented national school closures in March 2020. In the following months, many countries re-opened schools for face-to-face teaching with varying non-pharmaceutical interventions (NPIs) in place, such as reduced class sizes, staggered class start and end times, increased hygiene measures and use of face coverings [1]. However, subsequent waves of COVID-19 in many countries and ensuing lockdowns to limit transmission, have resulted in repeated or sustained school closures. School closures have the potential to lead to major adverse impacts on children and are likely to widen inequalities in educational attainment, often with lifelong impacts.

Children are less affected by COVID-19, compared to adults [2]. According to data from 29 countries, the proportion of children among COVID-19 cases varies from 0.3% (lowest in Spain) up to 13.8% (highest in Argentina) [3]. Evidence on SARS-CoV-2 transmission from children to other children and to adults in schools can support decision-making on the need for closure and re-opening of educational facilities during times of high community transmission and can inform mitigation measures in these settings. We are regularly updating a living systematic review on the evidence of SARS-CoV-2 transmission in school settings. Given the rapid pace of ongoing research, we aim to include new studies as they become available and to re-evaluate the conclusions. This review updates our previously published review with studies up to November 2020 [4].

## METHODS

### Protocol

The review protocol was developed following the reporting guidance in the Preferred Reporting Items for Systematic Reviews and Meta-Analyses Protocols (PRISMA-P) statement [5]. Protocol was registered on PROSPERO (register number: CRD42020192839) and was updated with new inclusion and exclusion criteria on 5 March 2021 [6].

### Literature search and eligibility criteria

We searched MEDLINE, CINAHL, ERIC, Embase, WHO COVID-19 database, medRxiv on 26 November 2020 with entry date limits from December 2019 (please see search strategies in Appendix S1 of the **Online Supplementary Document**), to identify studies that investigated SARS-CoV-2 transmission in schools. We performed parallel review of titles, abstracts, and subsequently full texts based on updated inclusion and exclusion criteria following the population, exposure, comparison, outcome (PECO) approach (according to the latest PROSPERO protocol registered). We included children (defined as ≤18 years old) who were attending school, and their close contacts (family and household members, teachers, school support staff). We excluded home-schooled children and their close contacts, and schools with student numbers below 20. For study outcomes, we included infections traced to a school index case with a COVID-19 positive test. We updated inclusion criteria of study types to include: 1) Cohort studies: A. Prospective cohort study: contact tracing study where the exposed contacts are followed up and secondary infections are measured. Secondary attach rates are (ideally) compared with another ‘community’ of unexposed participants matched for age and school to establish whether the school environment contributed to the secondary attack rate. B. Retrospective cohort study: positive cases in schools are identified through registries or contact laboratory databases, and then the contact tracing records scrutinized to assess the exposure and location of contacts and resultant rates where these were collected prospectively. These studies are less likely to have a comparison group. 2) Cross-sectional studies: A. Measurement of antibodies in a sero-surveillance study of schools at a point in time and then compared to background community rates at the same time. B. Measurement of active infection in all children/staff in a school with PCR or antigen tests at a single point in time after schools open (within the first 14 days of opening) and then comparing the infection rate with age-adjusted community rates before schools opening. 3) Pre-post studies, where community rates of acute infection are compared for a period before schools opening and two or more weeks after schools opening.

We excluded household studies unless specifically linked to school outbreaks; and studies where rates are measured in schools and without comparison of community rates. We included articles in peer-reviewed journals and pre-prints, and excluded comments, conference abstracts and interviews.

### Data extraction

Data relevant to the evidence for SARS-CoV-2 transmission in schools were extracted independently by two reviewers (WX, YD). Data included: citation details, publication type, study design, country, region, city, investigation period, background population setting (country/regional COVID-19 prevalence rates), types of non-pharmaceutical intervention in the background population setting, school closures at the time of the study, number of schools included, type of schools, size of schools, types of non-pharmaceutical interventions in place in schools, sampling method (nasopharyngeal or oropharyngeal swabs/ serum samples), provider testing vs self-testing, testing method (PCR/ SARS-CoV-2 antibody testing), modality of follow-up, frequency of follow-up, case and contact demographics (age and gender), clinical characteristics, number of index cases, number of contacts, number of secondary infected cases, IAR: No. of secondary infected cases/ No. of contacts, number of participants tested for SARS-CoV-2, number of SARS-CoV-2 positive cases, and SARS-CoV-2 positivity rates: No. of positive cases/ No. of participants tested.

### Meta-analysis

We pooled SARS-CoV-2 infection attack rates (IAR) or positivity rates using a random-effects model (DerSimonian-Laird) [7]. To account for zero cell counts, we transformed raw numbers/proportions with the Freeman-Tukey double arcine method to stabilize the variance [8]. Heterogeneity among studies was tested using Cochran's Q statistic, the *I^2^* index, and the tau-squared test [9]. Funnel plots and the Egger test were used to detect evidence of publication bias [10]. *P* < 0.05 was considered as statistically significant (two-sided).

### Risk of bias assessment

Two independent reviewers (NS, CM) evaluated the risk of bias in the included studies using the Newcastle Ottawa Scale (NOS) for controlled cohort and cross-sectional studies modified to reflect the school setting [11] and informed by earlier work [12]. The tools included an assessment of selection, measurement and attrition bias, and comparability, and considered how well the study performed compared to an idealised comparative study of school vs community rates. The tool is available in the supplementary materials (Appendix S2 of the **Online Supplementary Document**).

All statistical analyses were conducted using R, version 3.3.0 (R Foundation for Statistical Computing).

## RESULTS

### Characteristics and quality of the included studies

**Figure 1:**
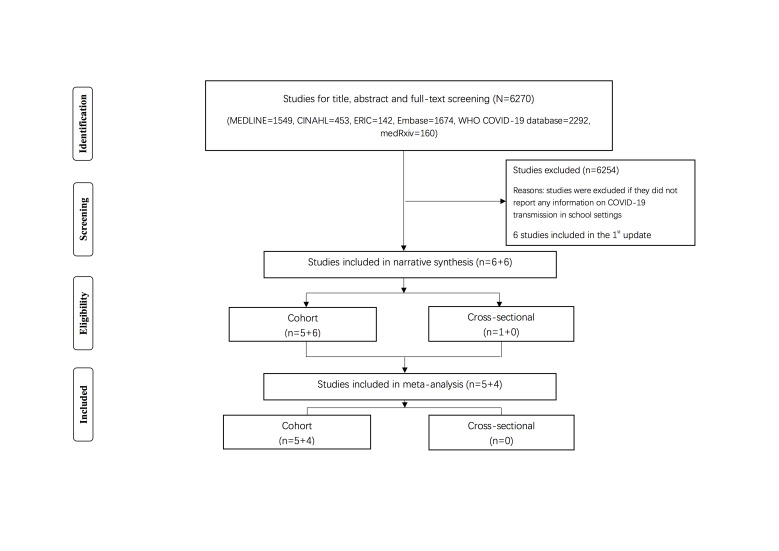
Flowchart summarizing study identification and selection.

6270 articles were retrieved from the systematic search. Based on our new inclusion and exclusion criteria 12 studies were retained in our review: 1^st^ update: n = 6 (cohort studies [13-17] and cross-sectional studies [18-23]; 2^nd^ update: n = 6 (cohort studies [24-29]) (**Figure 1**) and the characteristics of the included studies are presented in **Tables 1-4**. We found 11 cohort studies [13-17,24-29] that investigated secondary infection attack (**Table 1** and **Table 2**) and one cross-sectional studies [18] that investigated SARS-CoV-2 positivity compared to community rates (**Table 3** and **Table 4**). Five cross-sectional studies [19-23] from our 1^st^ update that investigated SARS-COV-2 positivity but did not compare with community rates were excluded.

**Table 1:** Characteristics of cohort studies (N = 11)

Study	Publication type	Study design	Country	Region	City	Investigation period	No. COVID-19 cases (background population)	Non-pharmaceutical interventions (country/region)	School closures (Yes/ No)	School closures (date)
**First update (n = 5)**										
Danis-2020 [13]	peer-review	cohort	France	Rhne-Alpes	Les Contamines-Montjoie	24 Jan-16 Feb	9	NA	Yes	8 Feb
Heavey-2020 [14]	peer-review	cohort	Ireland	NA	NA	1-13 Mar	90	NA	No	NA
Yung-2020 [15]	peer-review	cohort	Singapore	NA	NA	Feb-Mar	1189	NA	No	NA
NCIRS-2020 [16]	pre-print	cohort	Australia	New South Wales	NA	10 Apr-3 Jul	437	NA	10-28 Apr: Yes; 29 Apr-3 Jul: No	10-28 Apr
Macartney-2020 [17]	peer-review	cohort	Australia	New South Wales	NA	25 Jan-9 Apr	2779	NA	No	NA
**Second update (n = 6)**										
Lopez-2020 [24]	peer-review	cohort	United States	Utah	Salt Lake County	1 Apr-10 Jul	13943	NA	No	NA
Link-Gelles-2020 [25]	peer-review	cohort	United States	New England	Rhode Island	1-31 Jul	101	NA	No	NA
Brown-2020 [26]	peer-review	cohort	United States	NA	NA	10-13 Mar	240	NA	No	NA
Larosa-2020 [27]	pre-print	cohort	Italy	Reggio Emilia province	NA	1 Sep-15 Oct	6336	NA	No	NA
Dub-2020 [28]	pre-print	cohort	Finland	The Greater Helsinki region	Helsinki	Mar	95	NA	No	NA
Ehrhardt-2020 [29]	peer-review	cohort	Germany	Baden-Württemberg	NA	25 May-5 Aug	453	NA	No	NA

NA – not available

**Table 2:** Characteristics of cohort studies (N = 11)

Study	No. schools	Type of schools	Size of schools	Non-pharmaceutical interventions (school)	School cluster outbreak (Yes/ No)	Sampling method	Provider testing/ self-testing	Testing method	Follow-up modality	Follow-up frequency	No. index case	Type of Index case	Age	Gender	Contacts (N)	Secondary infected cases (n)	IAR (%)
**First update (n = 5)**																	
Danis-2020 [13]	3	NA	NA	school closed	No	nasopharyngeal swabs; endotracheal aspirates	NA	real- time RT-PCR	telephone call	daily	1	pupil	9	NA	102	0	0.00
Heavey-2020 [14]	NA	NA	NA	NA	No	NA	NA	NA	NA	daily	6	3 pupils; 1 staff; 2 adult visitors	pupils: 10-15; staff: >18	NA	1155	2*	0.17
Yung-2020 [15]	3	2 preschool; 1 secondary school	NA	terminal cleaning of schools; suspension of extracurricular, sport activities; staggered recess breaks	No	nasopharyngeal swabs	provider testing	real- time RT-PCR	NA	NA	3	2 pupils; 1 staff	pupils: 5, 12; staff: >18	NA	119	0	0.00
NCIRS-2020 [16]	6	3 primary school; 2 high school; 1 ECEC	NA	NA	No	nasopharyngeal swabs; serum samples	provider testing	nucleic acid testing; SARS-CoV-2 antibody testing	NA	NA	6	4 pupils; 2 staff	pupils: <18; staff: >18	NA	521	0	0.00
Macartney-2020 [17]	25	15 primary and secondary school; 10 ECEC	NA	NA	Yes	nasopharyngeal swabs; serum samples	provider testing	nucleic acid testing; SARS-CoV-2 antibody testing	text message; telephone call	NA	27	12 pupils; 15 staff	pupils: 14 (1-18) †; staff: 38 (19-65) †	pupils: 6 male, 6 female; staff: 1 male, 14 female	1448	18	1.24
**Second update (n = 6)**																	
Lopez-2020 [24]	3	childcare centre	NA	daily temperature and symptom screening; frequent cleaning; staff mandatory masks	Yes	nasopharyngeal swabs	provider testing	RT-PCR	NA	NA	3	staff	>18	NA	162	19	11.73
Link-Gelles-2020 [25]	29	childcare centre	20 persons per child care	limit to 20 persons; masks for adults; daily symptom screening; cleaning and disinfectant	Yes	NA	provider testing	RT-PCR	phone call; text	phone call (weekly); text (daily)	52	30 pupils; 20 staff; 2 visitor	pupils: 5 (0.5-12) †; staff: 30 (20-63) †	pupils: 14 male, 16 female; staff: 1 male, 21 female	853	17	1.99
Brown-2020 [26]	1	high school	NA	quarantine	Yes	serum samples	provider testing	ELISA antibody testing	NA	NA	1	staff	>18	NA	21‡	1	4.76
Larosa-2020 [27]	36	8 Infant-toddler centre and preschool; 10 primary school; 18 secondary school	NA	mandatory masks; single desks 1m apart; suspended extra-curricular activities; temporal and spatial pathways for different classes	Yes	nasopharyngeal swabs	provider testing	NA	NA	NA	43	38 pupils; 5 staff	pupils: <18; staff: >18	NA	1198	39	3.26
Dub-2020 [28]	2	NA	NA	NA	Yes	nasopharyngeal swabs; serum samples	provider testing	RT-PCR; MNT; FMIA	NA	NA	2	1 pupil; 1 staff	pupils: <18; staff: >18	NA	140	8	5.71
Ehrhardt-2020 [29]	11	3 childcare centre; 1 primary school; 4 secondary school; 3 vocational school	NA	group sizes reduced by 50%; cleaning of contact surfaces; regular and interim ventilation of rooms; exclusion of sick children; individual hygiene (hand hygiene, cough etiquette); face mask outside classroom; physical distancing	Yes	nasopharyngeal swabs	provider testing	NA	NA	NA	6	pupils; staff	pupils: <18; staff: >18	NA	NA	15	NA

ECEC – early childhood education and care setting, IAR – infection attack rate, NA – not available

*In other transmission settings (household, recreactional activities), except school settings.

†Median (range).

‡120 pupils that were in contact with the infected teacher and only 21 were tested. This study is not eligible for meta-analysis.

**Table 3:** Characteristics of cross-sectional studies (N = 1)*

Study	Publication type	Study design	Country	Region	City	Investigation period	No. COVID-19 cases (background population)	Non-pharmaceutical interventions (country/region)	School closures (Yes/ No)	School closures (Date)
**First update (n = 1)**										
Stein-Zamir-2020 [18]	peer-review	cross-sectional	Israel	Judean Highlands	Jerusalem	18 May-30 Jun	8863	NA	No	NA

NA – not available

*Studies investigated and cross-assessed SARS-CoV-2 positivity rates in schools and communities: n=1.

**Table 4:** Characteristics of cross-sectional studies (N = 1)

Study	No. schools	Type of schools	Size of schools	Non-pharmaceutical interventions (school)	School cluster outbreak (Yes/ No)	Sampling method	Provider testing/ self-testing	Testing method	Follow-up modality	Follow-up frequency	No. index case	Type of Index case	Age	Gender	Participants (N)	SARS-CoV-2 positive cases (n)	Positivity rate (%)
**First update (n = 1)**																	
Stein-Zamir-2020 [18]	1	high school	1352	daily health reports; hygiene; facemasks; social distancing; minimal interaction between classes	Yes	NA	provider testing	real- time RT-PCR	NA	NA	2	pupil	<18	NA	1312	178	13 · 57

NA – not available

#### Cohort studies (2^nd^ update)

We identified six new cohort studies in United States, Italy, Finland, and Germany that reported SARS-Cov-2 transmission in schools [24-29].

A cluster outbreak in schools was reported in Salt Lake County, Utah, United States during 1 April-10 July [24]. Three child care facilities had three SARS-CoV-2 positive index cases in school staffs attending while infectious, with 162 contacts traced. Secondary transmission was reported and infected cases were found in 12 children and 7 school staffs. IAR was estimated as 11.73%.

The Rhode Island Department of Health (RIDOH) in the United States conducted investigations of a reported COVID-19 case present at a child care program during June 1-July 31 [25]. Secondary transmission was reported in four childcare programs and with 52 positive index cases (children: n = 30, 58%; adults: n = 22, 42%), which resulted in closures of 89 classes and quarantine of 687 children and 166 staff members. 17 secondary infected cases were identified and the IAR was estimated as 1.99%. Despite limited evidence for secondary transmission, the impact on childcare programs was substantial, with 853 children and staff members quarantined

In the United States, a teaching staff member taught 16 classes while symptomatic, and of the 120 contacts including 48 (40%) enrolled in interactive classes, 72 (60%) enrolled in noninteractive classes were identified [26]. However, only 21 (18%) students participated in serologic survey during the quarantine period. Positive results were reported for 1 student (4.76%). Although this study indicates the risk associated with the classroom contact, the study is subject to limitations because of low participation.

A study in Reggio Emilia province, northern Italy investigated SARS-CoV-2 transmission in preschool and school settings after school reopening during September 1-October 15 [27]. In this study, 43 index cases among 38 students and 5 teaching staffs were reported. Thirty-nine secondary cases (3.90%) were identified among 994 children tested, in a total of 13 classes: in one primary school, and 8 secondary schools. The attack rate was higher in secondary schools (6.64%) than in primary schools (0.44%), while there were no secondary cases in the preschool settings. There were no secondary cases among tested teachers and staff members.

In Helsinki, Finland, incidents in two different schools were reported in March [28]. In school A, the index case was a student and no secondary infections occurred. In school B, one school staff led to eight (16%) secondary cases which were found in 51 close contacts.

A study investigated SARS-CoV-2 transmission in children aged 0 to 19 years old in childcare facilities and schools after school reopening in Baden-Württemberg, Germany during 25 May-5 August [29]. A total of 15 students were infected, 11 of which were infected from student-to-student and four infected from teaching staff-to-student. The study suggests that child-to-child transmission in schools and childcare facilities is uncommon and not the primary cause of SARS-CoV-2 infection in children.

#### Cross-sectional studies (2^nd^ update)

We did not find any new cross-sectional studies meeting inclusion criteria that investigated and compared positivity rates in schools and communities.

### SARS-CoV-2 infection attack rate

**Figure 2:**
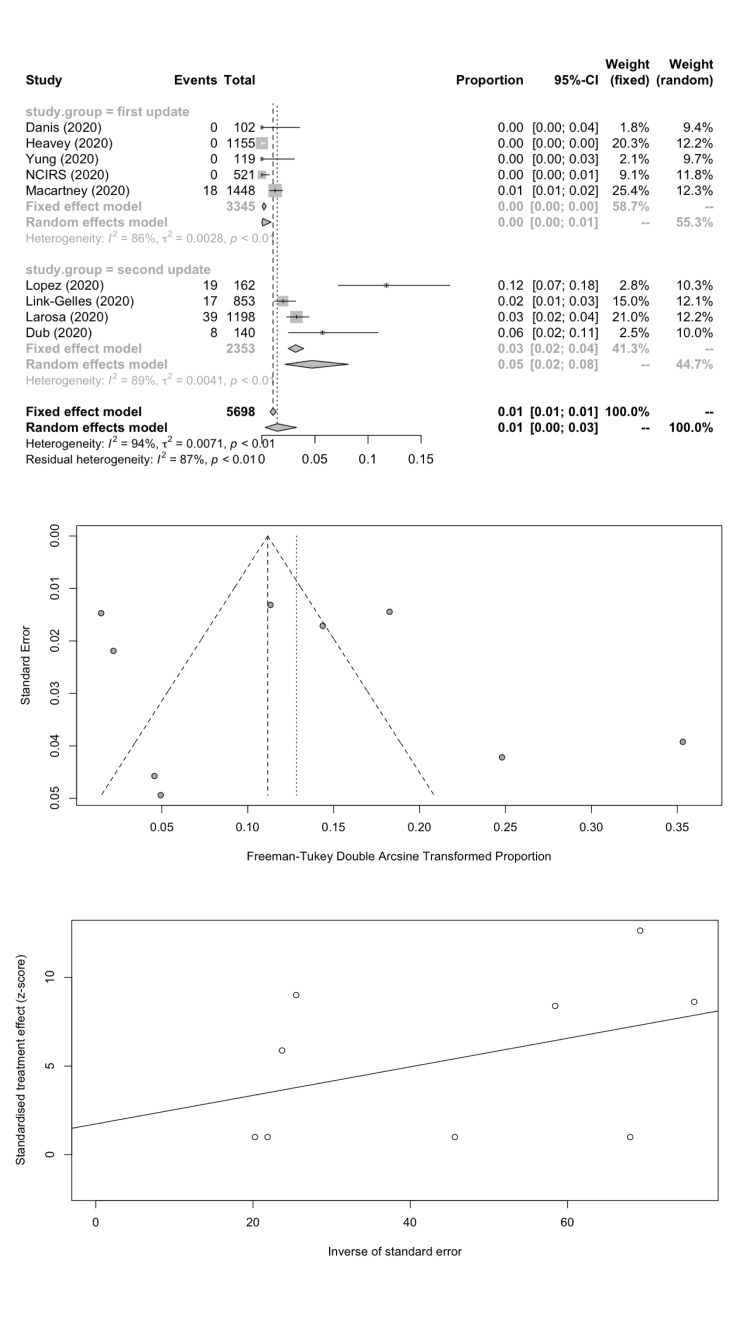
Overall infection attack rate. **Panel A.** Forest plot. **Panel B.** Funnel plot. **Panel C.** Egger’s plot.

We combined SARS-CoV-2 IARs in schools in a meta-analysis (**Table 5**). A total of nine cohort studies (1^st^ update: n = 5; 2^nd^ update: n = 4) were included with 101 secondary infected cases in 5698 contacts. The remaining two studies did not report the number of contacts. The pooled IAR of total study participants was calculated to be 1.45% (95% CI = 0.31%-3.26%) by using the Freeman-Tukey double arcine transformation and DerSimonian-Laird random-effects model (**Figure 2**, panel A). The heterogeneity in this meta-analysis was substantial with an *I^2^* value of 94.3%. There was no evidence of publication bias (Egger’s test *P* = 0.625; **Figure 2**, panels B and C).

**Table 5:** SARS-CoV-2 infection attack rate meta-analyses results

	Number of studies	n (infected cases)	N (contacts)	IAR (%)	95% CI	Cochrane Q	*I^2^*	*Tau*-square	*P*-Egger
Total	9	101	5698	1.45	0.31-3.26	139.84	94.3	0.0071	0.6249
Student	5	61	3645	1.66	0.08-4.78	106.25	96.2	0.0095	0.5852
School staff	5	15	704	1.18	0.00-4.43	23.91	83.3	0.0095	0.9612

CI – confidence interval, IAR – infection attack rate

**Figure 3:**
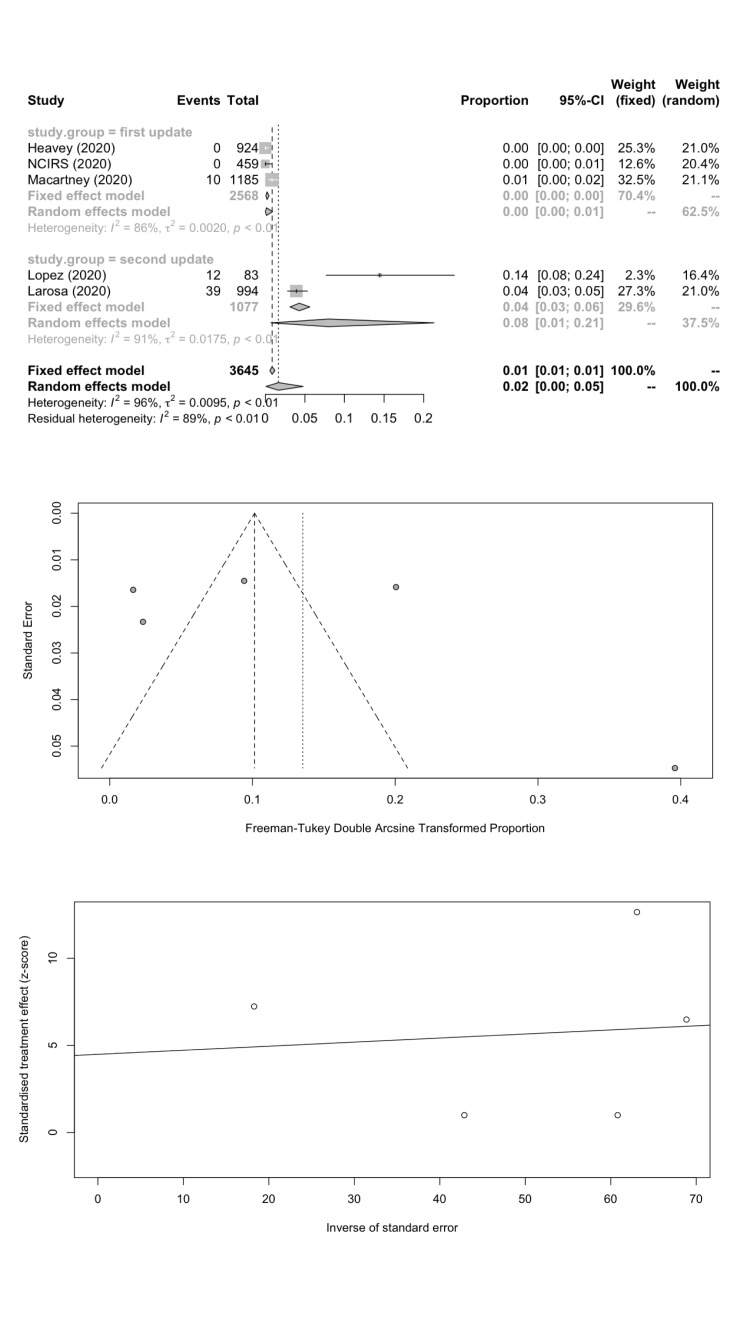
Student infection attack rate. **Panel A.** Forest plot. **Panel B.** Funnel plot. **Panel C.** Egger’s plot.

**Figure 4:**
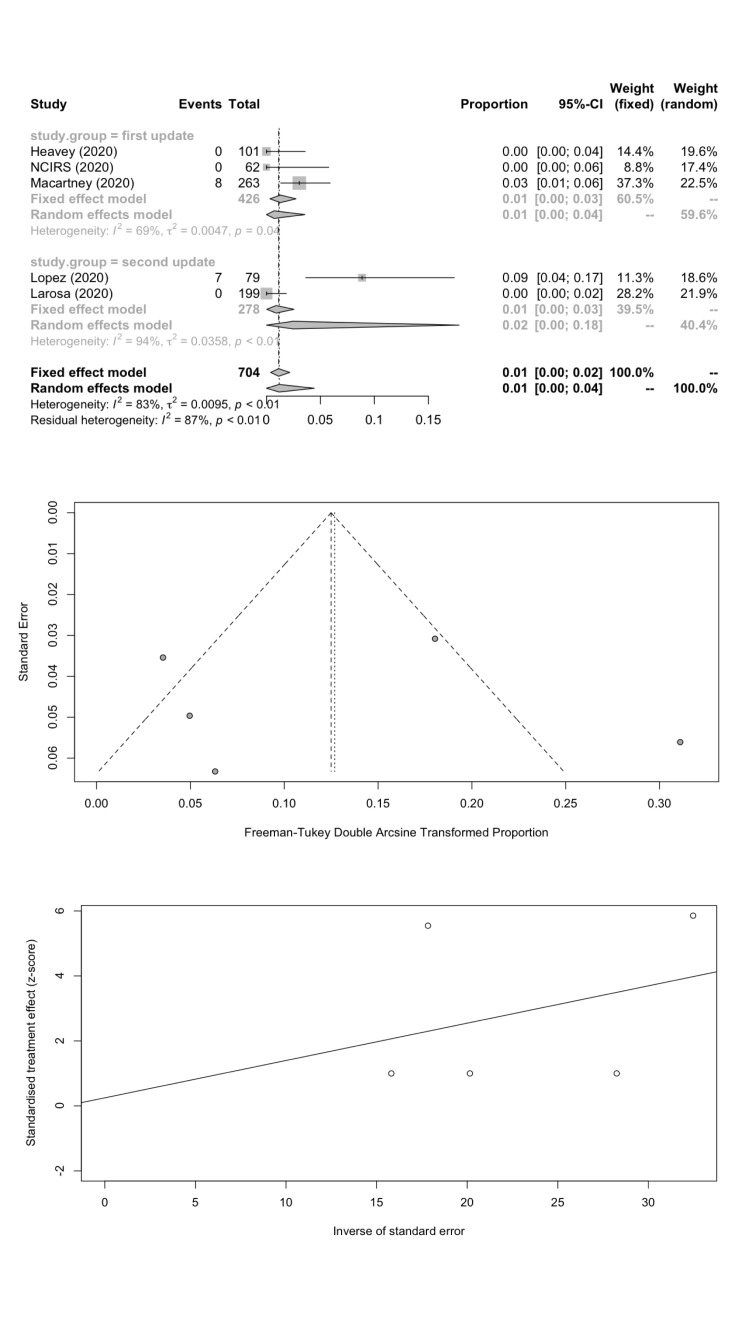
School staff infection attack rate. **Panel A.** Forest plot. **Panel B.** Funnel plot. **Panel C.** Egger’s plot.

We estimated the pooled IARs for students and school staff separately: 1.66% (95% CI = 0.08%-4.78%) and 1.18% (95% CI = 0.00%-4.43%) respectively; **Figure 3**, panel A; **Figure 4**, panel A). Heterogeneity was high and there was no evidence of publication bias (**Figure 3**, panels B and C; **Figure 4**, panels B and C).

### SARS-CoV-2 positivity rate

We only found one study (in 1^st^ review update) [18] which compared positivity rates in schools and communities, and therefore we could not conduct the meta-analysis to quantify the influence of school opening on SARS-CoV-2 transmission from the school settings to the communities. The high school outbreak in Jerusalem, Israel reported an overall IAR of 13.57%, with 153 students (attack rate: 13.18%) and 25 staff (attack rate: 16.56%) who were COVID-19 positive. As school reopened, the positivity rate of schoolchildren in the community increased to 40.93% (316/772) in weeks 22-25.

### Risk of bias

For cohort studies, all studies were at risk of selection bias to varying degrees. Although most studies performed well in terms of representativeness of the exposed group in the school, all but one performed poorly for representativeness of the unexposed groups, and no studies confirmed that the outcomes were not present at the start of the study. Comparability was reasonable across all studies with most schools matched for NPI measures and participants matched for age. Ten studies were at high risk of detection bias caused by differences in screening or testing or both, and 9 of 11 studies were at high risk of attrition bias with loss-to-follow up more than 20% or not described in the 14-day follow-up period (**Table 6**).

**Table 6:** Quality assessment based on modified Newcastle-Ottawa scale for cohort (contact tracing) studies

Study ID	Selection bias				Comparability		Detection bias		Attrition bias	
**Representativeness of exposed group**	**Representativeness of unexposed group**	**Ascertainment of exposure**	**Outcome not present at start of study**	**Matching for school mitigation policies**	**Matching for age**	**Assessment of SARS-CoV-2**	**Confirmation of SARS-CoV-2**	**Adequacy of length of follow-up**	**Loss-to-follow-up rate**
Brown 2020 [26]	–	*	*	–	*	*	*	*	*	*
Danis 2020 [13]	*	–	*	–	*	*	–	–	*	–
Dub 2020 – Incident A [28]	*	–	*	–	*	*	–	–	*	–
Dub 2020 – Incident B [28]	*	–	*	–	*	*	–	–	*	*
Erhardt 2020 [29]	*	–	–	–	–	*	–	–	–	–
Heavey 2020 [14]	*	–	–	–	*	*	–	–	*	–
Larosa 2020 [27]	*	–	*	–	*	*	*	–	*	–
Link-Gelles 2020 [25]	*	–	–	–	*	*	–	–	*	–
Lopez 2020 [24]	–	–	–	–	*	*	–	–	*	*
Macartney 2020 [17]	*	–	**	–	*	*	–	–	*	–
NCIRS 2020 [16]	*	–	*	–	*	*	–	–	*	–
Yung 2020 [15]	*	–	*	–	*	*	–	*	*	–

An ‘*’ denotes that the study met the criteria; a ‘–‘ denotes either that the study did not meet criteria or that it was not clearly reported.  For *Ascertainment of Exposure* two * can be awarded if both structured interviews and school review of timetables were undertaken.

A single cross-sectional study assessed SARS-CoV-2 positivity in schools and communities. The study was at risk of performance and detection bias (**Table 7**).

**Table 7:** Quality assessment based on modified Newcastle-Ottawa scale for cross-sectional studies

Study ID	Selection bias		Performance bias			Detection bias			Attrition bias	Comparability	
**Representativeness of sample**	**Percentage participation**	**Ascertainment of COVID-19**	**Confirmation of COVID-19**	**Blinding of assessors to prior exposure**	**Ascertainment of exposure to SARS-CoV-2**	**Confirmation of exposure to SARS-CoV-2**	**Blinding of assessors to COVID-19 status**	**Percentage in final analysis**	**Comparable in school**	**Comparable in age**
Zamir 2020 [18]	*	*	*	NR	NR	*	NR	NR	*	*	*

*Denotes that the study met the criteria; NR denotes either that the study did not meet criteria or that it was not clearly reported.

## DISCUSSION

This living systematic review summarizes the most recent evidence to understand SARS-CoV-2 transmission in schools and includes a study quality assessment to aid interpretation. The results from cohort and cross-sectional studies found that the overall IAR and SARS-CoV-2 positivity rate in school settings are low. Our previous review suggested lower IAR and SARS-CoV-2 positivity rate in students compared to school staff [4], whereas in this update when we combined studies from the second and first update we found higher IAR in students when compared to school staff.

Cohort studies estimated the secondary infection attack rates in school settings. Compiling the data from nine studies (EU countries: n = 6; United States: n = 2; Asian country: n = 1), we report an overall IAR of 1.45% (95% CI = 0.31%-3.26%) [13-17,24,25,27,28]. Cluster outbreaks were identified in five of the nine (55.6%) reporting countries, however, those that occurred were limited in number and size, varied from 0.01% (lowest in 15 primary and secondary schools, 10 ECDC in NSW) to 0.12% (highest in three childcare centers in United States) [17,24,25,27,28]. In addition, students reported higher IAR than school staff, which indicates that students were more susceptible to get infected through close contact with index cases. However, there is uncertainty about which grade school children are more likely susceptible to and transmit SARS-CoV-2 in schools. IARs for ECDC (early childhood education and care setting) (<6 years old), primary school (6-12 years old), and secondary school (12-18 years old) were 2.25%, 0.92%, 0.00% respectively in NSW. By comparison, the attack rate was higher in secondary schools (6.64%) than in primary schools (0.44%), while there were no secondary cases in the preschool settings, in northern Italy. The data are limited to reach a consensus. In addition, the studies of the school clusters in NSW, United States, and northern Italy demonstrated that factors related to physical distancing and face masks may play a role in the transmission of SARS-CoV-2 in school settings. Therefore, we suggest effective implementation of NPIs such as physical distancing, small-size class, cancellation of school mass gatherings, hand hygiene, and wearing of face masks, with school re-opening [1].

Cross-sectional studies estimated the proportion of SARS-CoV-2 positive cases, to provide an indication of how many people have been infected in schools. The SARS-CoV-2 positivity rates in the general study population (student/staff) under school environment varied from 0.00% (lowest in eight daycare centers in Belgium) to 25.87% (highest in one high school in France) [18-23]. Furthermore, the lower positivity rate was found in students, which suggested that students are less susceptible to infection and/or less frequently infected than adult school staff. Our finding is in line with previous studies comparing sero-prevalence between children and adults [30-33]. In general, the majority of countries report slightly lower seroprevalence in children than in adult groups, however these differences are small and uncertain. In Chile (Santiago), SARS-CoV-2 positive rates for pre-school (<6 years old), primary school (6-12 years old), secondary school (12-18 years old) were 12.24%, 10.84%, and 8.85% respectively. The peak rate was observed in pre-school. The sero-positivity was also higher (3.8%) in grades 1-2 (6-9 years old) in Switzerland (Zurich). By comparison, SARS-CoV-2 positive rates were higher in secondary schools in France (38.33%), Israel (13.18%). In addition, few cross-sectional studies cross-assessed SARS-CoV-2 positivity rates in schools and in communities, to investigate the impact of school opening on transmission. We suggest future research could compare positivity rates to evaluate whether SARS-CoV-2 is more easily to spread in school environment. Furthermore, we suggest large-scale sero-surveillence studies to monitor SARS-CoV-2 infection during school opening and schools could respond quickly to outbreaks with monitoring.

The main strength of this living systematic review is that it estimates pooled IARs and SARS-CoV-2 positivity rates for students and school staff, and it is updated with new studies to re-evaluate the conclusions given the rapid pace of ongoing research, to investigate the rate of SARS-CoV-2 transmission in schools. In addition, our study provides a critical assessment of the evidence, to aid the understanding of SARS-CoV-2 transmission risk in the school environment. However, the following potential limitations should be considered. First, further interpretation of age-group differences in IARs and positivity rates could not be performed because most of the included studies did not provide the raw data of student ages and we could not unify different age groups to run the meta-analysis. However, we suggest future studies could conduct sensitivity analyses for transmission rates in different school grades (child care/primary/sary) because this could provide evidence for the decision making of school closure and re-opening, with staggered class start and end times. Second, cross-comparisons between IARs and positivity rates reported in different regions/countries is difficult because of differences in the sampling and testing methods used, timing of the studies in relation to the outbreak, response measures and underlying community transmission. Moreover, the differences may contribute to the heterogeneity observed in the meta-analyses results and raise methodological concerns around the validity of the meta-analysis. Due to the limited number of included studies, we could not conduct subgroup meta-analyses to further investigate the heterogeneity. Third, seven studies in the included 12 studies (58.3%) reported prevention and control measures in place in schools such as physical distancing, face masks, class size, staggered class start and end times, and regular and interim ventilation of rooms, making it difficult to assess the effectiveness of NPIs under the school environment and to verify the argument whether transmission rates in schools could be reduced with effective NPIs in place. Forth, only one cross-sectional study has compared positivity rate in schools and communities to assess the impact of school opening on transmission from schools to communities. We additionally searched for study location background sero-prevalence or SARS-CoV-2 case rate per 100 000 population online, however, the data are currently limited. We suggest future studies could investigate this more focused research question: is school attendance associated with an increase or decrease in transmission in the community? Fifth, 25.0% (3/12) of included studies are pre-print publications and have not been peer-reviewed. For the majority of included studies there is high risk of bias and we should interpret the results with caution.

In conclusion, the balance of evidence so far indicates that the overall IAR and SAR-CoV-2 positivity rate in the school environment is low. Higher IAR were found in students compared to staff. Given the lack of clear evidence it will continue to be important to implement effective NPIs such as physical distancing, hand hygiene and smaller sized classes where possible to prevent schools from becoming a setting for accelerating onward transmission during the re-opening of schools.

## Additional material


Online Supplementary Document
